# Prostate Cancer Survivors: Physical, Emotional and Practical Concerns from the LIVESTRONG Survey

**DOI:** 10.3934/publichealth.2016.2.216

**Published:** 2016-04-13

**Authors:** Prajakta Adsul, Oussama M Darwish, Sameer Siddiqui

**Affiliations:** 1. Department of Behavioral Sciences and Health Education, College of Public Health and Social Justice, Saint Louis University; 2. Division of Urology, Department of Surgery, School of Medicine, Saint Louis University

**Keywords:** prostate cancer, survivorship, outcomes

## Abstract

**Objective:**

To determine whether a relationship exists between types of treatment received and/or survivorship status of prostate cancer survivors with physical, emotional, and practical concerns that they experience with the hypothesis that no such relationship exists.

**Methods:**

We analyzed data from the 2010 LIVESTRONG survey for cancer survivors which queried their physical, emotional, and practical concerns. This previously tested survey was administered between June 20, 2010 and March 31, 2011 on the LIVESTRONG.org website. Survivorship status was categorized as reported by the respondents: currently on treatment; living with cancer as a chronic condition; finished treatment less than 1 year ago; 1–5 years ago and; more than 5 years ago. Four categories were established for the types of treatment received: surgery, radiation, hormonal, and combination therapies. One-way ANOVA's were conducted to detect differences between groups and descriptive statistics were reported.

**Results:**

Of 2,307 respondents overall, only 281 males were included in this study based on self-reported primary diagnosis of prostate cancer and US residency status. The mean age of respondents was 60 years (SD = 8.54 years) and majority were white (90%). One-way ANOVA detected significant differences between the number of physical (*p* = 0.02), emotional (*p* = 0.04), and practical (*p* = 0.00) concerns for patients receiving different treatments. When compared across the survivorship trajectory, only number of practical concerns (*p* = 0.00) experienced by prostate cancer survivors were significantly different.

**Conclusions:**

Study findings highlight significant differences in number of concerns experienced by the patients based on their survivorship stage and the type of treatment received. Incorporating strategies to address the differences in physical, emotional, and practical concerns are essential to help physicians and clinical team members provide high quality post treatment survivorship care.

## 1. Introduction

In the last 25 years, the 5-year relative survival rate for all stages of prostate cancer has increased from 67.8% to 99.7%.[1] As of January 1, 2014 approximately 3 million prostate cancer survivors were estimated to be living in the United States and nearly 200,000 new cases would be diagnosed in that year.[2] Statistics also show that many men are being over-diagnosed and a significant number of these men are being treated aggressively, where no treatment would be necessary.[3]–[6] Many of these survivor's deal with long term reduction in functional outcomes such as urinary incontinence, sexual function and bowel function.[7] In addition, several prostate cancer survivors also experience clinically relevant general distress, increased anxiety and nearly 10% suffer major depressive disorder.[8],[9]

As patients continue to live longer following treatment, they are vulnerable to various late effects that may impact function, activities of daily living, and overall quality of life. There are few studies that have been published about how best to manage and care for survivors facing a myriad of not just physical but emotional and mental health challenges.[10] Kent et.al. analyzed the health information needs of cancer survivors from two cancer registries in California and showed a 60% prevalence of interpersonal and emotional information needs among cancer survivors.[11] A recent survey reporting the unmet needs of prostate cancer survivors in the United Kingdom showed that although patients reported high levels of satisfaction with the overall care, it was lower in relation to how well their emotional, psychological, relationship related and social problems had been addressed when compared with physical problems.[12] Qualitative work with cancer survivors has suggested that they have specific emotional and physical needs that require input from both, medical and non-medical caregivers.[13]

A better understanding of concerns that prostate cancer survivors experience can help health care professionals develop better strategies for their post treatment survivorship plans.[14] Although experiencing cancer-specific concerns is fairly common, survivors (especially men) may be embarrassed or ashamed to mention these concerns to members of their health care team.[15] Studies assessing quality of life (QoL) among prostate cancer survivors have outlined the significant side-effects that a patient with advanced prostate cancer may sustain and indicate how QoL assessment tools may contribute to care management and delivery.[15] In fact measuring and identifying issues of QoL can create an opportunity for the patients to discuss problems and receive information from health professionals.[11] Survivorship care with a patient-centered approach, which includes responding to patients' needs, effective communication, information sharing, encouraging adoption of healthy lifestyles and assistance accessing community services is essential to reduce this evidence gap. Many survivors however, do not report even a discussion with providers about psychosocial effects of cancer.[16] This reflects a missed opportunity in delivering supportive care for survivors that could have unmet emotional and psychosocial needs.

Several studies have evaluated the quality of life among prostate cancer survivors and shown comparison with either the type of treatment or the length of treatment [17]–[19]. There is however, limited research on identifying the psychosocial and practical needs among American prostate cancer survivors. Many of the measurements used (for example, Health Related Quality of Life (HRQOL) Scale [17]) do not provide a comprehensive understanding of a patient's quality of life which includes emotional, physical and practical concerns. Furthermore, there is mixed evidence on whether the physical, practical and emotional concerns vary over time since treatment. For example, Lubeck et al. conclude that among the 692 men enrolled in CapSURE database, the HRQOL scores were stable over time with patients undergoing observation, radiotherapy and or hormonal therapy [17] whereas Miller et al. report that the HRQOL scores remained stable for prostatectomy but continued to evolve in men treated with radiotherapy [20]. Therefore, the objective of this study was to comprehensively investigate the physical, emotional, and practical concerns experienced by prostate cancer patients. We also wanted to describe whether there were any differences in these concerns depending on the type of treatment received or the length of survivorship. Based on the literature review, we hypothesized that there would be no relationship between the concerns and the type of treatment received and/or the length of survivorship.

## 2. Methods

The data available from the 2010 LIVESTRONG survey provided us the opportunity to test our hypothesis. The target population for this survey were individuals who had been diagnosed with cancer, who had currently finished treatment or managing cancer as a chronic condition which included those still taking medication to prevent a recurrence as well as those still seeing a doctor to check for new or returning cancers. The LIVESTRONG foundation invited people who have been impacted by cancer and those who have never been diagnosed, but have a personal connection to cancer, such as having a loved one who has been diagnosed to answer the survey. However, the data analysis in the present paper only includes people who reported a primary diagnosis of prostate cancer and those that reported to be residents of the United States.

It is important to note that the 2010 LIVESTRONG Survey for Post Treatment Cancer survivors covers several areas identified as policy priorities in cancer survivorship research and care as identified by the Institute of Medicine [21] and the National Cancer Institute.[22] It was created in collaboration with cancer survivors and aimed at comprehensively assessing the physical, practical and emotional needs of cancer survivors. The survey instrument was developed using the Quality of Life in Adult Cancer Survivors (QLACS) and was cognitively tested with cancer survivors.[23] Further details about the development and validation of the survey can be found elsewhere. [24] The LIVESTRONG foundation made the online survey available to anyone who was interested in taking the survey; hence a response rate could not be calculated for the respondents. It was administered online between June 20, 2010 and March 31, 2011 and the overall report is available online at www.livestrong.org.[25]

The survey instrument was divided into five sections which covered 1) physical concerns, 2) emotional concerns, 3) practical concerns, 4) positive experiences with cancer, and 5) resources and information provided by LIVE**STRONG**. Only the first three domains were included for data analysis presented in this paper. The survey questions were designed to ask participants whether they had any of the physical, emotional, or practical concerns as a result of their experience with cancer at any stage of their survivorship including pre-, during and post-treatment. The extensive list of concerns also included items related to late effects of cancer and its treatment such as lymphedema, fatigue, sadness and depression, and issues with insurance.

A total of 38 statements were used to ascertain physical concerns, 31 for emotional concerns, and another 38 for practical concerns. Each statement was presented with the following text: “Since completing treatment, have any of the following statements been true for you as a result of your experience with cancer?” followed by the response options: “yes, ” “no, ” or “I don't know. ” All concerns were used for the analysis and are provided in Appendix A. In order to determine meaningful differences, the types of treatment received by the cancer survivors were separated into four categories: surgery, radiation, hormonal and combination therapies. Combination therapies could have included any of the above mentioned categories in addition to immunotherapy, biological therapy, bone marrow transplant, and peripheral blood stem cell transplant.

The survivorship status of the respondents was defined using the following five categories: currently on treatment; living with cancer as a chronic illness; finished treatment less than 1 year ago; finished treatment between 1-5 years ago and finished treatment more than 5 years ago. These categories were based on the self-report of respondents to the following question: “What is your current stage of survivorship?” The response options were as follows: (1) I am currently on treatment; (2) I am living with cancer as a chronic illness; (3) I finished treatment less than a year ago; (4) I finished treatment between 1–5 years ago; and (5) I finished treatment 5 or more years ago. The LIVESTRONG report included respondents that were still taking medication after their primary treatment to prevent a recurrence as well as those still seeing a doctor to check for new or returning cancers in the category of “living with cancer as a chronic illness”. Therefore, in terms of prostate cancer patients this category most likely included respondents who were on active surveillance or watchful waiting. None of the data used in this study was collected from patient records; demographic and clinical data were attained solely through patient self-report on the survey.

This paper presents a cross-sectional analysis of the data gathered from respondents. Given our research questions we had two specific *a priori* hypothesis: (1) There were no significant differences in the number of physical, emotional, and practical concerns across types of treatment received (surgery, radiation, hormonal and combination). (2) There were no significant differences in the number of physical, emotional, and practical concerns across the survivorship trajectory (currently on treatment; living with cancer; finished treatment less than 1 year ago; between 1-5 years ago; and more than 5 years ago). We tested our hypotheses using the ANOVA test of analysis of variance. Post hoc analyses were carried out to identify specific differences among the groups. In addition, we also conducted descriptive statistics on the physical, emotional, and practical concerns among the prostate cancer survivors. All data were analyzed using the Statistical Package for Social Sciences (SPSS version 20).

In the overall report that presented findings from 2,307 participants, the respondents were mostly white (92%) and female (67%), one to five years post treatment (61%) and college graduates (59%). Of these respondents, only 281 males were included in the analysis based on prostate cancer diagnosis and US residency status.

## 3. Results

Table 1 enumerates the characteristics of 281 respondents included in the study sample. Table 2 lists the most common physical, emotional and practical concerns that were surveyed by LIVESTRONG. A total of 38 physical concerns were presented in the survey with respondents indicating a mean of 6.98 (SD = 6.03) concerns. Thirty-one emotional concerns were included in the survey and respondents indicated a mean of 6.61 (SD = 6.61) concerns. Lastly, 38 practical concerns were included with respondents indicating a mean of 1.03 (SD = 1.2) concerns.

One-way ANOVA was conducted to detect differences in the number of physical, emotional and practical concerns across types of treatment received (surgery, radiation, hormonal and combination) and the survivorship status (currently on treatment; living with cancer as a chronic condition; finished treatment less than 1 year ago; between 1-5 years ago; and more than 5 years ago). As seen in Table 3, significant differences were seen in number of physical (*p* = 0.02), emotional (*p* = 0.04) and practical (*p* = 0.00) concerns for patients receiving different treatments. On post-hoc examination of the 95% Confidence Interval's (CI's) in Table 3, both physical and emotional concerns were less among men receiving radiation when compared to men that underwent surgery or combination therapies. The number of practical concerns were significantly less among men receiving combination therapies as compared to surgery.

**Table 1.** Characteristics of the study population

Study Sample Characteristics	Number (%)
**Age**	60 (8.54)
Mean (SD)	
**Race**	254 (90.1)
White	10 (3.5)
Hispanic	4 (1.5)
Black	14 (3.9)
Other	
**Survivorship status**	64 (22.7)
Currently on treatment	25 (8.9)
Living with cancer as a chronic illness	57 (20.2)
Finished treatment < 1 year ago	93 (33.0)
Finished treatment 1–5 years ago	37 (13.1)
Finished treatment > 5 years ago	5 (2.1)
Missing	
**Type of treatment**	133 (47.2)
Surgery	40 (14.2)
Radiation	6 (2.1)
Hormonal	102 (36.3)
Combination	

**Table 2.** Most common physical, emotional, and practical concerns as indicated by prostate cancer survivors

	Concerns	Total Count	Percent
	**Physical concerns**		
**1.**	I have been bothered by difficulty or inability to function sexually	198	70.2
**2.**	I have been dissatisfied by my sex life	165	58.5
**3.**	I urinate more frequently than I used to	151	53.5
**4.**	I have avoided sexual activity or lacked interest in sex	138	48.9
**5.**	I have had difficulties with impotence	114	40.4
**6.**	I have felt tired a lot	98	34.7
**7.**	I have had trouble getting the rest that I need	89	31.5
**8.**	I have not had the energy to do the things I wanted to do	87	30.8
**9.**	I have had trouble sleeping for several nights in a row	81	28.7
**10.**	I have not been able to control when I urinate	74	26.2
			
	**Emotional Concerns**		
**1.**	I have worried about cancer coming back	173	61.3
**2.**	I have felt grief about the death of other cancer patients	147	52.1
**3.**	I have worried about whether my family members might have cancer causing genes	143	50.7
**4.**	I have worried that my family members were at risk for getting cancer	139	49.2
**5.**	I have felt anxious	116	41.1
**6.**	I feel blue or depressed each time these dates or events occur	115	40.7
**7.**	I have worried about dying from cancer	103	36.5
**8.**	I have felt blue or depressed	101	35.8
**9.**	I have felt that I have lost a sense of security	88	31.2
**10.**	I have been bothered by mood swings	81	28.7
			
	**Practical concerns**		
**1.**	Due to a cancer diagnosis, I have spent above and beyond my insurance	253	89.72
**2.**	Due to a cancer diagnosis, I have debt	122	43.26
**3.**	My loved ones or I have had financial problems because of cancer, treatment or late effects of cancer	22	7.80
**4.**	I am unable to work in the same way I did before	16	5.67
**5.**	Since completing my treatment, I have not been able to get the prescriptions I needed because of my health insurance	16	5.67
**6.**	Since completing my treatment, I have problems with health insurance because of cancer as a pre-existing condition	13	4.61
**7.**	I have had difficulty with the return to work	12	4.26
**8.**	I have stayed in my job because I did not want to lose my health insurance	11	3.90
**9.**	Since completing my treatment, I have not been able to get additional health insurance	6	2.13
**10.**	I left my job	5	1.77

**Table 3.** Association between type of treatment received and type of concerns for prostate cancer survivors

	Physical (0–38)		Emotional (0-31)		Practical (0-38)	
	Mean (SD)	95% CI	Mean (SD)	95% CI	Mean (SD)	95% CI
Surgery	7.00 (4.94)	6.16-7.84	6.75 (4.86)	5.92-7.58	1.31 (1.10)	1.13-1.50
Radiation	4.51 (5.39)	2.89-6.13	4.31 (5.18)	2.76-5.87	0.89 (0.88)	0.62-1.15
Hormonal	9.00 (6.16)	2.53-15.47	9.00 (6.99)	1.67-16.33	0	0
Combination	8.06 (7.28)	6.59-9.54	7.42 (6.56)	6.09-8.75	0.76 (1.43)	0.47-1.05
ANOVA	F (3,39.25) = 3.76, p=0.02*		F (3,25.81) = 3.04, p=0.04*		F (3,277) = 5.94, p=0.00*	

*significant at 0.05 level

When compared across survivorship groups, only practical concerns were significantly different within the groups (*p* = 0.00) as presented in Table 4. Respondents with missing information about survivorship status were excluded from this analysis. Also noted in Table 4, is that the mean number of practical concerns increase with time since prostate cancer treatment, whereas the mean number of physical and emotional concerns decrease. Post hoc tests revealed that prostate cancer patient's currently receiving treatment had significant differences in number of practical concerns compared to individuals in other survivorship groups.

**Table 4.** Association between survivorship stage and type of concerns for prostate cancer survivors

	Physical (0–38)		Emotional (0–31)		Practical (0–38)	
	Mean (SD)	95% CI	Mean (SD)	95% CI	Mean (SD)	95% CI
Currently on treatment	6.96 (7.18)	4.80–8.39	5.34 (5.99)	3.85–6.84	0.06 (0.04)	−0.01–0.14
Living with cancer	8.96 (8.10)	5.62–12.30	9.04 (6.62)	6.31–11.77	0.32 (0.69)	0.04–0.60
Finished treatment < 1 year ago	7.33 (5.37)	5.91–8.76	6.77 (5.54)	5.30–8.24	1.30 (1.10)	1.01–1.59
Finished treatment 1–5 years ago	6.58 (5.10)	5.53–7.63	7.02 (5.17)	5.96–8.09	1.51 (1.12)	1.27–1.74
Finished treatment > 5 years ago	6.68 (5.60)	4.81–8.54	6.24 (5.76)	4.32–8.17	1.57 (1.71)	1.00–2.14
ANOVA	F (4,134.48) = 0.79, p = 0.53		F (4,271) = 2.12, *p* = 0.08		F (4,111.14) = 22.45, *p* = 0.00*	

*significant at the 0.05 level

## 4. Discussion

Prostate cancer survivors face several challenges during and after treatment. The data presented in this study highlight a significant association between the patient’s survivorship trajectory and the type of treatment received with the number and type of concerns experienced as reported by the respondents to the LIVESTRONG survey. Understanding the relationship between physical, emotional, and practical concerns and their treatment can help physicians and their clinical team members plan and incorporate strategies to address them throughout the patient’s health care experience.

The most commonly reported physical concerns as indicated by the survey respondents were related to their sexual and urinary dysfunction. These side-effects are often directly related to the type of treatment that the patient receives. Hormonal treatments can cause significant sexual side effects [26] and as reported in Table 3, the number of physical concerns among the respondents that received hormonal treatment were highest at mean of 9.00 (SD = 6.16). Hormonal therapy is most often indicated in prostate cancer patients that present with advanced or metastatic disease[27]. It is likely that these patients also experience additional physical problems including bone pain, fatigue, and hot flashes which could explain the high rate of physical concerns in patients receiving hormonal therapy. It is important to note however, that this group also showed the greatest variability in their confidence intervals suggesting that respondents indicated either very low or high numbers of physical concerns (CI: 2.53–15.47). In addition to the physical concerns, the number of emotional concerns was also significantly related to the type of treatment received and was highest among patients receiving hormonal therapy. This may be related to the fact that a majority of the hormonal patients suffer from metastatic disease and are dealing with a cancer in a manageable but incurable state. It may also be related to the hormonal treatment itself, as androgen deprivation therapy is shown to be associated with clinically diagnosed depression.[28] Again, it is important to note that this group also had wide CI's from 1.67 to 16.33.

As noted in Table 4, the mean number of physical and emotional concerns decrease with time since prostate cancer treatment. As time passed, it could be possible that survivors were able to get some help for their physical concerns (e.g. sexual and urinary function). Consistent with other studies [29], the most common (n = 173) emotional concern identified by prostate cancer survivors in this study was the fear of cancer recurrence. Patients also worried about their family member's susceptibility to cancer (n = 143) along with psychological issues such as feeling anxious (n = 116) and depressed (n = 101). Several systematic reviews [30],[31] have noted no significant change over the survivorship trajectory which is in contrast with the respondents in our survey who reported less emotional concerns over the survivorship trajectory. However, one study did note that younger age was consistently associated with greater fear of recurrence while older patients reported less fear.[32] It could be possible that the survey respondents who were further out from treatment were older and therefore reported less emotional concerns in the data presented in this study.

In contrast to the emotional and physical concerns, the respondents in the survey reported increased practical concerns. This was also noted in Table 4, where the practical concerns increased as the patient's reported being further away from treatment. The present study provides a candid view of the practical concerns that were identified by prostate cancer survivors. Financial concerns were identified by the prostate cancer patients as the most common practical concerns. A systematic review about the information needs and sources of information among cancer patients in general, identified only 4 studies that described financial support needs of cancer patients such as cost of treatment and insurance coverage among other issues.[33] Estimates based on claims data have calculated average monthly costs of up to $2,187 for prostate cancer patients when compared to $329 among individuals without cancer.[34] A recent Irish study exploring the financial and economic burden of a cancer diagnosis on patients and their families revealed a wide range of cancer-related medical and non-medical expenses, a major impact on work productivity and household income along with difficulties in accessing medical care benefits.[35] There is enough evidence to show that the economic burden associated with cancer is substantial and can partly explain the increased practical concerns experienced by the respondents in our survey further out from treatment. However, there is limited evidence for an accurate assessment of these issues among prostate cancer survivors and even less so regarding appropriate interventions for addressing these issues. Similar studies in the context of the American healthcare system are required to understand the unmet needs of the prostate cancer survivors.

The study findings have at least two immediate implications. First, there is an immediate need for routine assessment of patients' psychological well-being not just before or during treatment but also during the post treatment survivorship period. Second, findings also suggest the need for including health professionals such as psychologists in the survivorship care plans. There is emerging evidence that engaging in support groups can increase the quality of life for prostate cancer survivors by providing access to shared experiences, resources for guidance in decision making, involvement in group education and discussion, psychosocial and spiritual support, and support the long term adoption and maintenance of healthy behaviors. [36] Support groups can play an important role in managing the emotional needs of prostate cancer patients and further research to explore the role of support groups is warranted.

The present study has a few limitations that need to acknowledged. First, it is a cross sectional study of the prostate cancer survivor's perceptions at a given point in time. These perceptions are susceptible to change over the course of their survivorship and their access to health care. Since our study was cross-sectional in nature, no inferences could be made on the trends of these concerns as respondents were different in each group studied. More research is required to further examine this trend in a longitudinal study with prostate cancer survivors.

Second, data presented in this paper is representative of mostly white men with the ability to access computers and answer online surveys. Based on the final report of the LIVESTRONG 2010 survey, overall a total of 2,307 individuals were included in the final dataset. The majority of the respondents were white (92%) and female (67%), one to five years post treatment (61%) and college graduates (59%). When compared to the Surveillance Epidemiology and End Results program (SEER) databased of the National Cancer Institute in the US, there were many notable differences.[25] For example, the SEER database provides a 20% prevalence of prostate cancer however, only 12% of the LIVESTRONG survey respondents were prostate cancer survivors. This may be partially attributed to the fact that the survey was entirely administered online and not many survivors may be internet savvy. Nonetheless, respondents included in this analysis may not be entirely representative of the actual population needing post treatment supportive care in the US. However, this data does give some insight into the needs of the survivors; enough to warrant further research into the unmet needs of the prostate cancer survivors.

Lastly, findings from this data rely on patient self-report of their symptoms, experiences and clinical characteristics. Approximately 9% of the study population identified themselves as “living with cancer as a chronic illness.” We hypothesized that in the case of prostate cancer, these patients were on active surveillance or watchful waiting. It is possible that since the data included in this study is self-reported, findings may suffer from volunteer and/or recall bias.

Despite the recent Institute of Medicine report highlighting the need for post cancer follow up care [37], cancer survivorship care remains poorly coordinated not only during treatment but also after receiving treatment. The primary debate in the current literature seems to be about who should provide survivorship care between the oncologists and the primary care physicians.[38] However, findings of this study showcase that the cancer survivors have several concerns which may require a team approach to implement a “shared care model” to deliver optimal quality of care. The American Cancer Society prostate cancer survivorship care guidelines, address health promotion, surveillance for prostate cancer recurrence, screening for secondary primary cancers, long term and late effects assessment and management, psychological issues and care co-ordination among the oncology team, primary care clinicians and non-oncology specialists.[10]. Due to limited published evidence however, the guidelines do not incorporate plans or strategies tailored to the post treatment survivor's stage or the type of treatment received. In conclusion, findings from this study strongly suggest the need to tailor and consequently maximize our efforts on specific groups of prostate cancer survivors while providing overall clinical follow up care.
